# A Case Report of Fedratinib-Associated Uveitis

**DOI:** 10.7759/cureus.52373

**Published:** 2024-01-16

**Authors:** William Evans, Christina Mathew, James Richardson-May, Rashi Arora

**Affiliations:** 1 Ophthalmology, Salisbury District Hospital, Salisbury, GBR

**Keywords:** adverse drug event, hypopyon, drug induced uveitis, myelofibrosis, fedratinib

## Abstract

Drug-induced uveitis is a rare but important subgroup of uveitis particularly among newer drugs in the market. Establishing a diagnosis can be challenging and requires the physician to have a high index of suspicion and a holistic approach with consideration being afforded to history, clinical examination, and investigations. In this case report, we describe a case of hypopyon uveitis in a 64-year-old male with a background of myelofibrosis for which he was started on fedratinib. A thorough history, negative investigation panel, and temporal association between the start of the drug and uveitis helped establish the diagnosis. A literature review showed no other published cases of uveitis secondary to fedratinib. While he could not be withdrawn from the drug, collaboration with the medical team enabled close monitoring and follow-up. He recovered following a course of steroids and remains under observation.

## Introduction

Myelofibrosis is a myeloproliferative neoplasm (MPN) characterized by clonal myeloproliferation, often due to Janus kinase 2 (JAK2) mutations in the individual [1].

Fedratinib (Inrebic; Bristol-Myers Squibb) is a newly developed oral JAK2 inhibitor that was approved by the US Food & Drug Administration (FDA) in August 2019 for the treatment of adult patients with intermediate‐2 or high‐risk primary or secondary myelofibrosis, based on the results of the JAKARTA studies [2-5]. This made fedratinib the second FDA-approved drug to treat myelofibrosis, following the approval of ruxolitinib in 2011 [6].

When considering ocular side effects of fedratinib, a comprehensive literature search indicated a case of orbital inflammatory syndrome in one patient that occurred bilaterally following the commencement of fedratinib and resolved upon stopping the medication [7]. However, there otherwise remains a lack of clinical cases for ophthalmic complications, with no cases describing uveitis as a complication of the drug. We describe a patient with hypopyon panuveitis as a side effect of fedratinib treatment.

## Case presentation

A 64-year-old male attended the ophthalmology acute referral clinic with a one-day history of a red, photophobic, painful left eye and a dramatic decrease in vision since morning. He had a background of myelofibrosis and was on fedratinib treatment. In addition, he had a history of primary angle closure glaucoma in the left eye with advanced field loss and primary angle closure in the right eye for which he had previous laser peripheral iridotomy and was taking preservative-free latanoprost and dorzolamide.

The best corrected visual acuity (BCVA) was 6/6 in the right eye and 5/60 in the left eye. The intraocular pressure (IOP) was 12 mmHg in the right eye and 20 mmHg in the left eye. Slit-lamp biomicroscopy revealed conjunctival hyperemia in the left eye with grade three anterior chamber cells along with hypopyon and posterior synechia. Fundal examination was limited due to the presence of vitritis. There was a left relative afferent pupillary defect (RAPD). Examination of the right eye was unremarkable.

**Figure 1:**
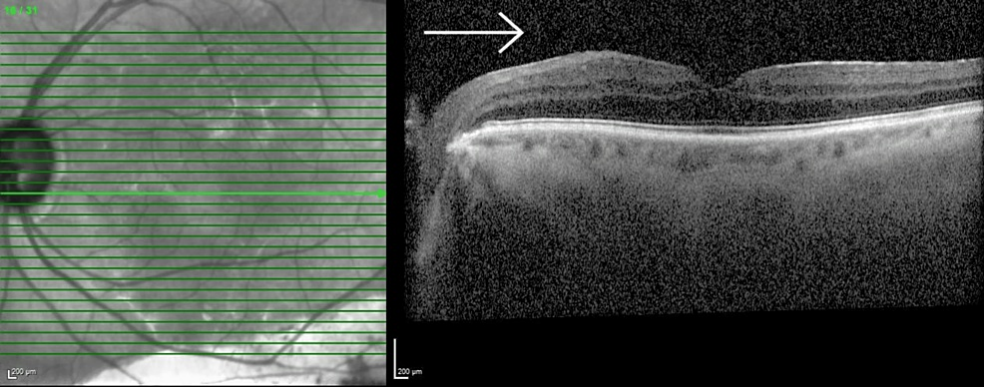
OCT of the left macula The image shows degradation of image quality as a result of vitreous opacities (marked), though no gross evidence of intraretinal fluid. OCT: Optical coherence tomography

**Figure 2:**
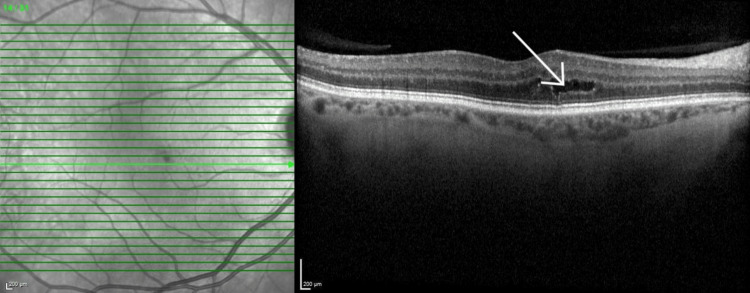
OCT of the right macula The image shows sharper image quality due to no inflammation. Incidental finding of outer retinal cyst is marked. OCT: Optical coherence tomography

Ultrasound B-scan showed a flat retina with the presence of vitreous opacities in keeping with vitritis. Ocular coherence tomography (OCT) showed widespread vitreous opacities but no evidence of macular edema. This is seen in Figure 1 for the left eye; the right eye is shown in Figure 2. 

Differential diagnoses

Uveitis can be due to a range of conditions, including infectious, inflammatory, and autoimmune causes. However, the vast majority are idiopathic. Indeed, the etiology for uveitis can be difficult to identify and necessitates the ophthalmologist to develop a holistic profile of the patient weaving together the history, examination, and investigations.

As such, extensive investigation was undertaken with laboratory and radiological tests. Blood tests including inflammatory markers, antinuclear antibody (ANA), anti-neutrophilic cytoplasmic autoantibodies (ANCA), angiotensin-converting enzyme, syphilis serology, Quantiferon, and HLA-B27 were negative, as was a plain film radiograph of the chest. In addition, there was a lack of underlying systemic symptoms; thus, it was felt that underlying causes such as sarcoidosis, Behcet’s, and Vogt-Koyanagi-Harada were unlikely [8].

The presence of hypopyon was concerning due to the possibility of endophthalmitis, either exogenous or endogenous. However, since there was no recent surgery and the patient had normal inflammatory markers and systemic symptoms were absent, this was ruled out [9]. In addition, hypopyon can occur with HLA-B27-associated uveitis as well as Behcet’s disease - in the latter, it is classically a “shifting” hypopyon [10].

With no systemic, autoimmune, or infectious causes identified, and given the temporal association between the commencement of uveitis preceded shortly by the commencement of fedratinib, it was inferred that the inflammation was most likely secondary to starting the new medication. Therefore, this was a diagnosis of exclusion wherein all potential causes were thoroughly investigated and excluded, while a thorough analysis of the patient’s history including medication history allowed us to elucidate the diagnosis.

Treatment, outcome, and follow-up

On presentation, he was started on hourly topical dexamethasone 0.1%, cyclopentolate 1%, and apraclonidine 1%. This was to continue alongside his regular glaucoma medication.

He was regularly reviewed over the following two weeks. A gradual improvement both subjectively and clinically was noted, with improvement in anterior chamber inflammation and consequently his visual acuity. Topical steroids were tapered over three months. He continued to improve, with no evidence of ongoing inflammation after 10 weeks. His visual acuity returned to his baseline visual acuity of 6/9.

**Figure 3:**
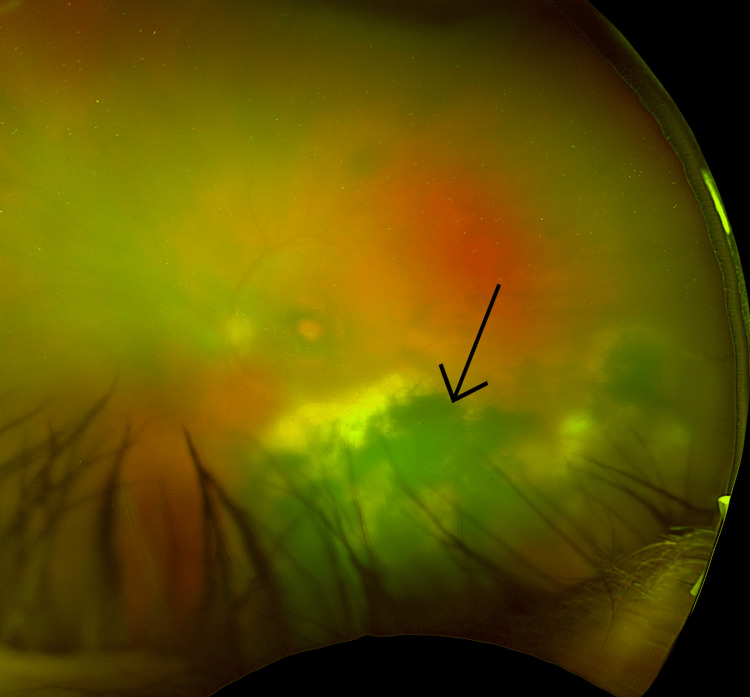
Optos image of left eye prior to treatment The image demonstrates previously known large inferior chorioretinal scar (marked) with significantly reduced image quality secondary to vitreous inflammation.

**Figure 4:**
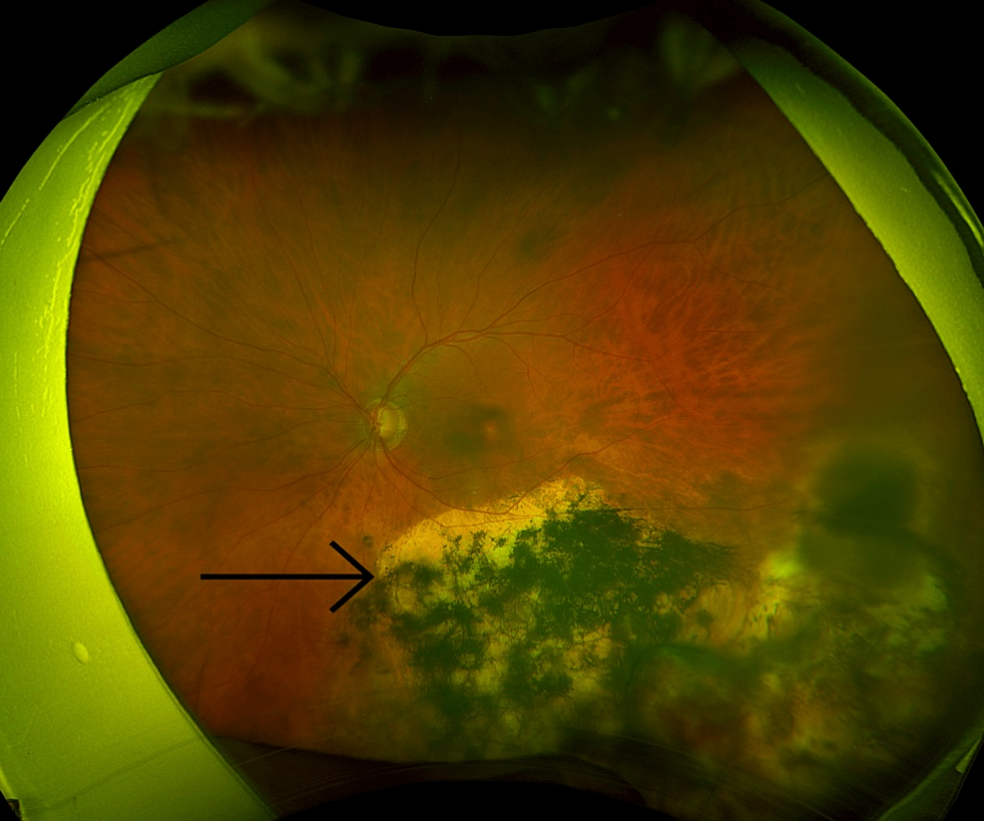
Optos image of left eye following treatment The image shows significant improvement in fundal view in keeping with resolution of inflammation, with the chorioretinal scar once again marked.

Optos retinal images of his left eye demonstrate the improvement in symptoms. Before treatment was commenced the image quality was significantly reduced due to vitreous inflammation, as can be seen in Figure 3. After successful treatment, the image showed a significant improvement in fundal view in keeping with the resolution of inflammation (Figure 4). 

He underwent cataract surgery once the eye was quiescent. Due to a steroid-related IOP rise, glaucoma control was difficult, but is being closely monitored with a plan to proceed with glaucoma drainage device surgery should IOP control remain difficult or the glaucoma progresses.

After a discussion with his medical team, it was agreed that as there was no alternative for fedratinib, this was to be continued, but with a plan for close collaboration with the ophthalmology department to ensure adequate follow-up and monitoring.

## Discussion

Whilst most cases of uveitis are idiopathic (48-70%), drug-induced uveitis is a rare entity accounting for less than 0.5% of all uveitis cases [11,12]. Establishing a diagnosis in these cases can often be challenging as it is a diagnosis of exclusion. Several criteria have been elucidated to establish drug-induced uveitis including resolution of uveitis on withdrawal of the drug, recurrence of uveitis on re-challenge with the drug, increased severity of uveitis at higher doses of the drug, and exclusion of other causes and similar cases of uveitis described with the drug or similar classes of the drug. However, rarely does a single drug meet all the criteria. The ophthalmologist must therefore maintain a high degree of suspicion coupled with thorough history, clinical exam, and investigations to establish the diagnosis. This is especially important when evaluating the newer drugs in the market for which drug-induced uveitis has not been reported in literature.

The pathogenesis of drug-induced uveitis is poorly understood but several theories have been postulated. Direct mechanisms are often associated with topical application and intracameral and intravitreal injections. Indirect mechanisms include hypersensitivity reactions secondary to immune complex deposition in uveal tissue, immune-induced reactions to antigens derived from drug-induced destruction of microbial agents, and alteration in the ability of melanin to scavenge free radicals [13].

In this instance, the patient was deemed to have panuveitis secondary to starting fedratinib, following a negative investigation panel and due to the temporal association between the commencement of the drug and the onset of symptoms. A comprehensive literature review showed no other published cases of uveitis secondary to fedratinib. However, other anti-cancer drugs such as immune checkpoint inhibitors and protein kinase inhibitors have a more recognized profile of drug-induced uveitis [14]. As this is a newer drug, it is vital that ophthalmologists are aware of the potential of this drug to cause uveitis and accordingly patients must be advised to report any symptoms suggestive of uveitis to their treating physician. Moreover, as a new drug with only one case of orbital inflammatory syndrome described in literature, it is important that ophthalmologists document other potential side effects of the drug in practice and report them via the Medicines and Healthcare products Regulatory Agency (MHRA) yellow card reporting system so that it can inform future practice.

Much like this case, most cases of drug-induced uveitis can be resolved with topical steroids, with more severe cases necessitating periocular and systemic steroids. Withdrawal of the drug often results in resolution of symptoms. However, this may not be a viable option for all patients. Thus, close collaboration between the ophthalmologist and treating physician enables prompt and appropriate management.

## Conclusions

In summary, while most cases of uveitis are idiopathic, it may also be secondary to autoimmunity, infection, drugs, or systemic disease. Establishing the diagnoses can be challenging and requires the ophthalmologist to exercise a high degree of suspicion, considering history, clinical examination, and investigations to correctly identify an etiology. However, this is crucial as eliminating the offending agent can often result in resolution of symptoms, and if uveitis is a presenting sign of another condition, early diagnosis enables prompt collaboration with other disciplines, leading to improved outcomes. This is the first reported case of uveitis induced by fedratinib. In addition to the previously reported orbital inflammatory syndrome, physicians must be aware of uveitis as a potential complication of this drug and advise patients accordingly. Early and close collaboration with ophthalmologists enables prompt treatment and monitoring. Furthermore, documenting and reporting additional cases in practice is crucial for expanding our understanding of this drug's potential complications.
